# Case of Severe Acidosis Recovering after Injection of Sodium Bicarbonate

**Published:** 1919-01

**Authors:** 


***A Case of Severe Acidosis Recovering after a Massive Intravenous Injection of Sodium Bicarbonate.*** By M. DESPLAS. *La Presse Médicale*, September 26, 1918.

A soldier wounded in the buttocks by a shell-fragment was in a very bad condition 13 hours after his injury: high temperature and arterial hypertension. He was very depressed and his condition resembled that of a shocked man, except for his ashen complexion, the arterial hypertension, and the fact that the local condition of the wound was excellent.

A case of acidosis was diagnosed, and immediate intravenous injection of 500 cc. of a solution of Sodium Bicarbonate at 50 per 1000, resorted to.

This injection was followed by gradual subsiding of temperature and hypertension, and improvement of the general condition.

Three days later the patient was considered as wholly out of danger.

Meanwhile the examination of the urine proved positive for the reaction of the diacetic acid (Gerhard reaction with iron perchloride).

This case of acidosis was not connected with any gangrenous infection, contrary to the generally admitted opinion.

